# Advancing our understanding of monocyte HLA-DR, S100A9, and the potential for individualized therapies in sepsis

**DOI:** 10.1186/s40779-023-00465-9

**Published:** 2023-06-25

**Authors:** Guillaume Monneret

**Affiliations:** 1 Hospices Civils de Lyon, Immunology Laboratory, Hôpital E. Herriot, 69437 Lyon, France; 2 Université de Lyon, EA 7426 “Pathophysiology of Injury-Induced Immunosuppression”, Université Claude Bernard Lyon-1, 69437 Lyon, France

**Keywords:** Sepsis, Monocyte, HLA-DR, S100A9, Immunosuppression

By definition, sepsis refers to a life threatening organ dysfunction due to a dysregulated host response to an infection [1]. More precisely, sepsis triggers a multifaceted response characterized by a simultaneous manifestation of pro-inflammatory and anti-inflammatory elements that disrupt mechanisms intended to maintain homeostasis. Initially, an overwhelming hyperinflammatory reaction ensues, resulting in tissue damage and organ dysfunction. In parallel, efforts to regulate the harmful excess of inflammation through negative feedback mechanisms can inadvertently result in a state of delayed immunodeficiency if not properly controlled in terms of timing and magnitude. This delayed immunodeficiency further heightens the vulnerability to nosocomial infections, increases the likelihood of rehospitalization, and even contributes to elevated long-term mortality rates [2]. By nature, the septic population is highly heterogeneous (e.g., with variations in comorbidities, the type of responsible pathogen, and the infected organ). This heterogeneity is further compounded by the huge variations in both intensity and temporal dynamics of individual immune responses in septic patients, posing challenges for immune interventions. In fact, the same therapeutic approach can yield both advantageous and detrimental effects depending on whether it is administered promptly or delayed. Undoubtedly, this overall heterogeneity has played a significant role in the prolonged lack of success in immunomodulatory therapies for sepsis, which was predominantly based on a too simplistic “one size fits all” approach [3].

To overcome this obstacle, there is currently a consensus on the need to improve the phenotyping of patients [4]. By defining distinct endotypes among patients, the objective is to identify individuals who are more likely to respond to a specific treatment approach (enrichment strategy) [5]. To accomplish this, it is crucial to integrate multiple facets of sepsis pathophysiology into a fully comprehensive understanding. Unfortunately, many valuable studies utilizing state-of-the-art technologies have often failed to transition into routine care because their results have not been integrated with the numerous disorders associated with sepsis. In contrast, the study conducted by Yao et al. [6], provides a comprehensive and integrated perspective on the progression of sepsis. The researchers employed single-cell RNA sequencing (scRNA-seq) to identify distinct subsets of monocytes (especially *HLA-DR*^*low*^*S100A*^*high*^*)* that exhibited temporal variations in different patient cohorts. These scRNA-seq findings were further validated in animal models of sepsis. Additionally, the team confirmed their results at the cellular level using flow cytometry and confocal microscopy. As low HLA-DR monocytes are believed to possess immunosuppressive properties, the authors next demonstrated through functional experiments based on sorted cells, their suppressive effects on T cells. Lastly and importantly, by administering Paquinimod [an inhibitor of S100A8/A9 proteins binding to Toll-like receptor 4 (TLR4)], they successfully reversed immunosuppression and improved the survival rate of septic mice. While the existence of immunosuppressive monocyte populations in sepsis is not entirely novel, the combination of various findings presented in this article constitutes a robust demonstration of the significant role played by this specific cell population in the outcomes of septic patients. The multi-faceted approach enhances the reliability and credibility of the main conclusion on *HLA-DR*^*low*^*S100A*^*high*^ monocytes. Beyond this compelling case, three novel notable results would deserve to be highlighted.

First, the authors highlight the importance of immune trajectories, including within the populations of monocytes themselves. They demonstrate that pro-inflammatory monocytes appear very early, followed by the emergence of suppressive monocytes. This illustrates the necessity of understanding the kinetics of biomarkers before considering their potential isolation at a specific time point [7, 8]. Unfortunately, this kinetic aspect is often neglected in clinical studies. It is essential to recognize that immunodeficiency induced by sepsis is a delayed process, and premature immunostimulation of patients could prove to be risky.

Secondly, the authors identify the intracellular protein S100A9 as a potential marker for the population of immunosuppressive monocytes. This finding is consistent with previous research, as S100A9 has been described as a characteristic marker of granulocytic myeloid-derived suppressor cells (G-MDSCs) [9]. While further confirmation is required, it is likely that the *HLA-DR*^*low*^*S100A*^*high*^ monocytes identified in this study are the monocytic counterpart of G-MDSCs [10]. Interestingly, S100A9 mRNA emerges as a biomarker of interest for identifying immunosuppressed patients [11, 12]. Recently, S100A9 mRNA has been shown to be part of a small panel of mRNA markers that can identify septic patients at risk of developing secondary infections [13].

Thirdly, the authors were able to reverse immunosuppression by blocking the binding of S100A9 to TLR4. While this aspect of the research may have some limitations in terms of controls, as acknowledged by the authors, it opens up new possibilities in understanding the pathophysiology and potential therapies for sepsis. It appears that there is a vicious circle wherein the bone marrow, following sepsis, produces cells containing high levels of S100A9. This, in turn, induces the generation of suppressive monocytes through the binding of S100A9 to TLR4. This process has also been proposed for other damage-associated molecular patterns (DAMPs) such as high mobility group protein 1 (HMGB1), nucleosomes, free DNA, and inflammatory cytokines [14]. In other words, chronic latent inflammation could partly contribute to persistent immunosuppression. This aligns with available clinical data [12]. However, this finding needs to be reconciled with the failure of anti-inflammatory strategies that have been attempted for decades. It may be worthwhile to reanalyze the designs of all clinical trials based on TLR4 antagonists in light of these new insights.

In conclusion, the findings presented in this study offer valuable knowledge that will contribute to a better understanding of sepsis, its endotypes, and the immune trajectories of patients. By identifying specific monocyte subsets, elucidating the role of S100A9, and highlighting the importance of immune kinetics, this research provides a solid foundation for developing more effective treatment approaches for sepsis. This paves the way for targeted and personalized therapies, which are increasingly within reach.

## Data Availability

Not applicable.

## Abbreviations

DAMPs: Damage-associated molecular patterns
G-MDSCs: Granulocytic myeloid-derived suppressor cells
HMGB1: High mobility group protein 1
scRNA-seq: Single-cell RNA sequencing
TLR4: Toll-like receptor 4
